# Structural Examination of 6-Methylsulphonylphenanthro-[9,10-C]-furan-1(3*H*)-one—A Rofecoxib Degradation Product

**DOI:** 10.3390/ph3020369

**Published:** 2010-02-01

**Authors:** Pamela M. Dean

**Affiliations:** Department of Chemistry, Monash University, Wellington Road, Clayton, Victoria, 3800, Australia; E-Mail: pamela.dean@sci.monash.edu.au; Tel.: +61-3-990-54599; Fax: +61-3-990-54597

**Keywords:** rofecoxib, structural characterisation, NSAID, crystallography

## Abstract

In the attempt to discover a new polymorph of rofecoxib (Vioxx®), an unexpected product resulted. The product was characterised by chemical composition, thermal behaviour and structure and found to be 6-methylsulphonylphenanthro-[9,10-C] furan-1(3*H*)-one, a photo-cyclization degradation product of rofecoxib. This is a significant finding because it indicates that without appropriate control of the recrystallisation procedures, the structural integrity of rofecoxib may be seriously compromised.

## 1. Introduction

**Figure 1:**
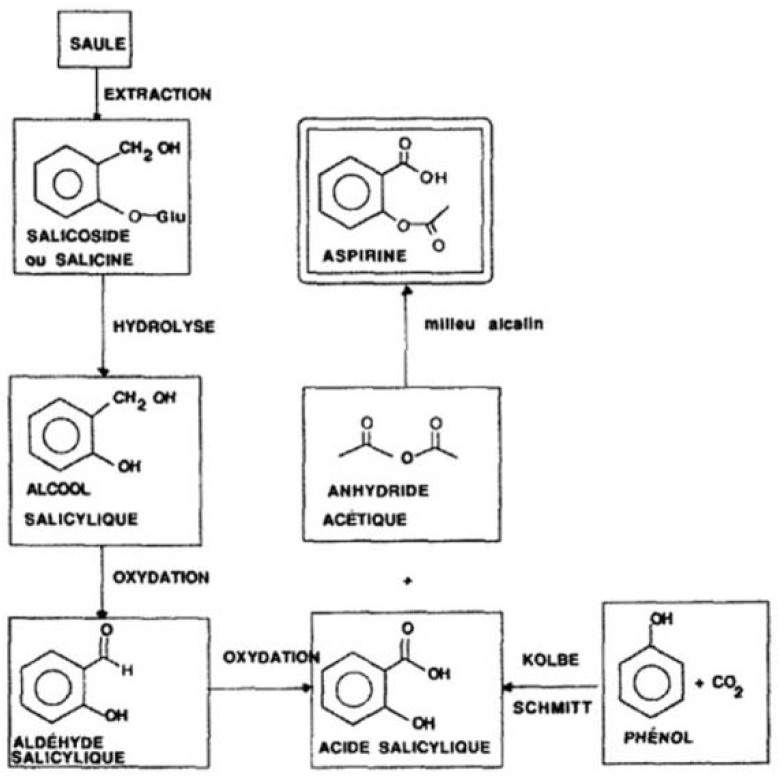
A synthetic route for acetylsalicylic acid by Hoffmann in 1897 [1].

The therapeutic properties of willow bark were known since antiquity, with the Egyptians and Assyrians using a decoction of myrtle and willow leaves to produce analgesia. Even Hippocrates, regarded as the father of medicine, recommended the chewing of willow leaves for this purpose. The active compound within willow bark was subsequently characterised and synthetically modified in 1875 by Felix Hoffman from the Bayer Company to form acetylsalicylic acid, commonly known as aspirin, (Figure 1) [1]. Subsequently in the 20^th^ century aspirin was discovered to exert in addition antipyretic and anti-inflammatory effects, thereby becoming the first known anti-inflammatory. Thereafter a plethora of anti-inflammatory drugs were synthesized, notably phenylbutazone, which was classed the first non-steroidal anti-inflammatory in 1949 [2].

**Figure 2:**
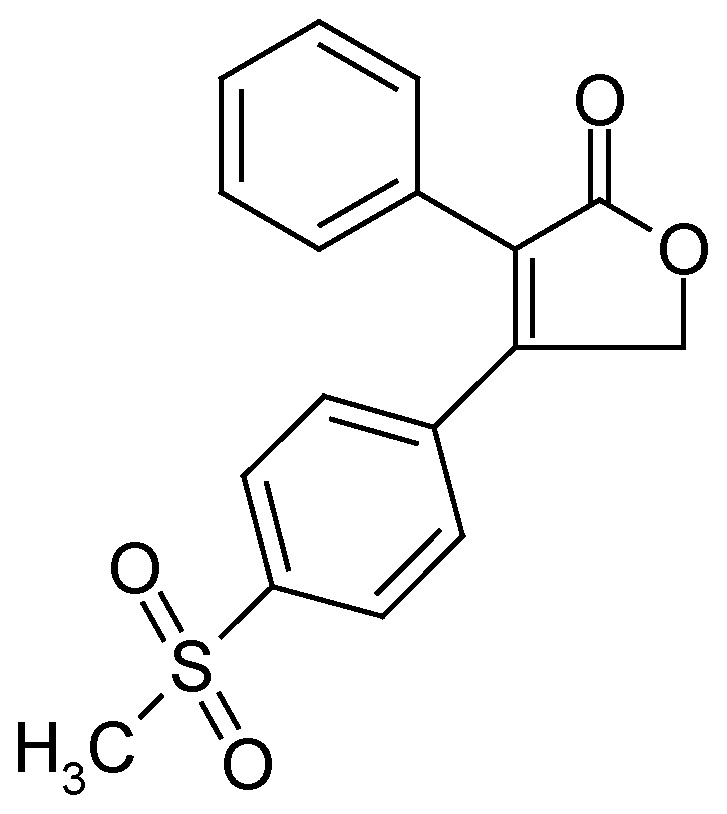
Structure of rofecoxib.

A recent non-steroidal anti-inflammatory drug used between 1999 and 2004 is rofecoxib [4-(4-methylsulphonylphenyl)-3-phenyl-2,5-dihydro-2-furanone, Vioxx®, Figure 2]. Rofecoxib is a Cox-2 inhibitor belonging to the diaryl substituted furanones. Rofecoxib has been used for the treatment of osteoarthritis, primary dysmenorrhoea and acute pain [3]. However, its effectiveness may be reduced due to its low solubility in water (~0.0086 mg/mL at 25 °C) and limited dissolution rate [4]. Therefore, to combat this problem different solid forms were pursued that might show superior solubility behaviour. However, in the attempt to discover a new polymorph, an unexpected product was obtained. This product, 6-methylsulphonylphenanthro-[9,10-C]-furan-1(3*H*)-one (**I**), resulted from two methods, recrystallisation and co-precipitation. Compound **I** has been previously reported in the literature, where its existence was mainly identified by standard chromatographic methods [5,6,7]. In these reports the degradation product was easily obtained by exposing a solution of rofecoxib-acetontrile or rofecoxib-acetonitrile/water to UV light. Herein, we report for the first time the existence and identification of **I** via standard crystallographic techniques.

## 2. Results and Discussion

### 2.1. Degradation Product Formation

**Figure 3:**
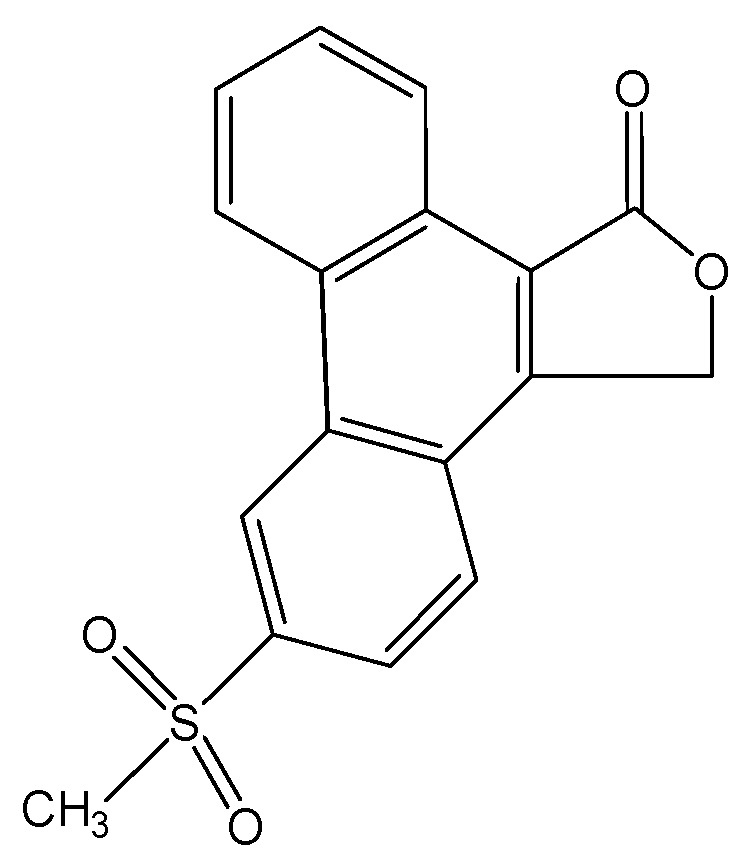
Structure of **I**.

Compound **I** resulted from two methods, recrystallisation and co-precipitation. In the recrystallisation experiment 10 mg of rofecoxib was dissolved in 2 mL of ethyl acetate and stirred at 65 °C for 15 min. For the co-precipitation experiment 10 mg of rofecoxib was stirred in 2 mL 10% HPβCD (hydroxypropyl-β-cyclodextrin) solution at 60 °C for 72 h. All solutions were exposed to room light intensity. The resultant solution was thereafter filtered and left to evaporate at room temperature. Crystal formed after one month. After testing, the product was identified as 6-methyl- sulphonylphenanthro-[9,10-C]-furan-1(3*H*)-one (**I**) [8] (Figure 3).

### 2.2. Thermal Analysis

**Figure 4:**
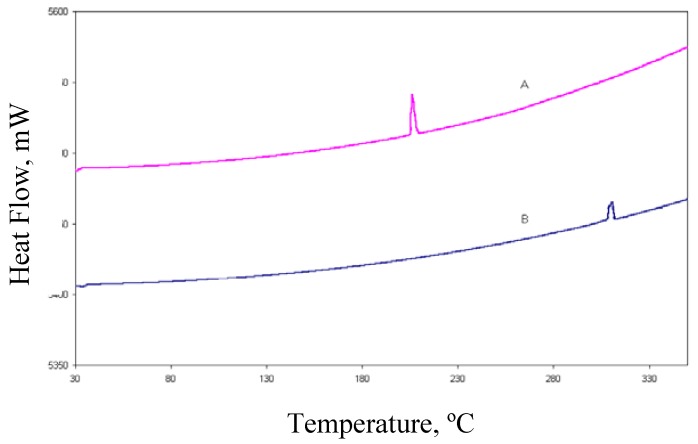
DSC curves for rofecoxib (A) and **I** (B).

DSC (differential scanning calorimetry) analysis revealed that rofecoxib exhibited a fusion endotherm (Figure 4) at onset temperature 209.3 °C (Δ*H_f_* = 125.0 J/g). Compound **I** also exhibited a single fusion endotherm but at the different onset temperature of 307.1 °C (Δ*H_f_* = 48.5 J/g) thereby implying that these are two different chemical species. TGA (thermogravimetric analysis) proved that both forms were free of solvent since there was a zero mass loss in the temperature range 30 °C–350 °C. 

### 2.3. X-Ray Crystallographic Analysis

#### 2.3.1. Unit Cell Determination

The unit cell parameters and crystal system of the new product were determined rapidly using the Nonius Kappa CCD diffractometer. A crystal of high quality was selected and mounted using Paratone N oil and used for data-collection. Table 1 lists the parameters determined for **I** in comparison to rofecoxib. The molecules within the crystal structure of rofecoxib are held together by van der Waals interactions and the crystal is tetragonal with space group P4_1_2_1_2 [9]. Compound **I** was structurally found to be a higher melting, cyclised degradation product of rofecoxib exhibiting three phenyl rings.

**Table 1:** Unit cell parameters of **I** and rofecoxib.

	I	Rofecoxib [9]
Space group	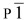	P4_1_2_1_2
a	8.4641 (1) Å	11.374 (2) Å
b	9.2247 (2) Å	11.374 (2) Å
c	9.4227 (2) Å	22.939 (3) Å
α	79.8987 (1) °	90 °
β	85.9977 (9) °	90°
γ	67.5502 (2) °	90°
Volume	669.41 (2) Å^3^	2967.6 (9) Å^3^
Z	2	8

#### 2.3.2. Structure Determination and Refinement

**Figure 5:**
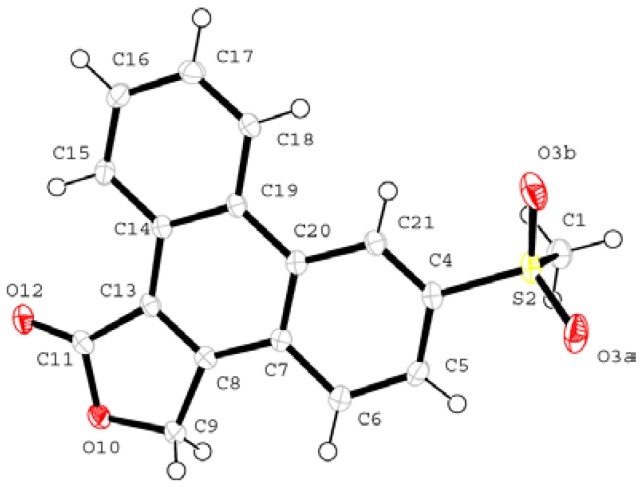
The asymmetric unit of **I** showing the numbering scheme (the hydrogen numbering has been omitted for clarity).

Direct methods yielded the positions of all the non-hydrogen atoms of the asymmetric unit. The atomic numbering scheme is shown in Figure 5. The hydrogen atoms are numbered according to their parent atoms to which they are bonded. Refinement was carried out with all of the non-hydrogen atoms treated anisotropically. The hydrogen atoms were located in subsequent difference electron density maps and placed in geometrically constrained positions and refined with isotropic temperature factors assigned at 1.2 times the U_iso _values of the parent atoms. 

#### 2.3.3. Geometrical Analysis

The rings are planar with a significant (0.114(3) Å) deviation of C6 above the plane of the phenyl ring and an equal deviation (0.114(2) Å) of C5 below the plane of the phenyl ring. The methyl-sulphonyl substituent is directed below the plane. All hydrogen bonding is of C-H…O type. These are listed in Table 2.

**Table 2:** Hydrogen bond data for **I**.

Donor --- H…Acceptor	Distance (Å)	Angle (°)
	D - H H…A D…A	D - H…A
C(1) --H(1A) …O(3B) ^i^	0.98 2.56 3.444(3)	150
C(15) --H(15) …O(12)^ ii^	0.95 2.54 3.168(2)	124
C(21) --H(21) …O(3B)^ ii^	0.95 2.50 2.904(2)	106

^i^ Related by symmetry operation: 1-x, 1-y, 1-z; ^ii ^Intramolecular bonding.

**Figure 6:**
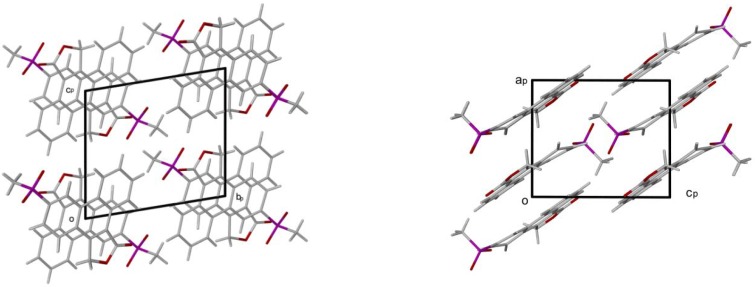
Packing diagrams of **I** down the *a*-axis and *b*-axis projections.

The other prominent interactions occurring are X--HLπ and π-π ring contacts. These are described in Table 3. Cg is the centre of gravity of the aromatic ring indicated by a numeral in the legends scheme. The packing of **I** is shown in Figure 6 clearly showing the π-π ring interactions and the orientation of the molecule to allow the hydrogen bonding.

**Table 3:** X--HLπ ring interactions for **I**. 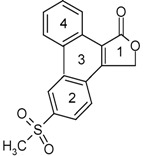

X--H…Cg (Pi-Ring) / Cg(A) … Cg(B)	Distance (Å) H…Cg X…Cg / Cg…Cg	Angle (°) X—H…Cg
C(9) --H(9B) …Cg(4) ^i^	2.681 3.447	134.4
Cg(1) … Cg(3)^ii^	3.554(1)	
Cg(1) … Cg(4)^ii^	3.724(1)	
Cg(2) … Cg(2)^iii^	3.812(2)	
Cg(2) … Cg(3)^iii^	3.813(1)	
Cg(3) … Cg(3)^ii^	3.596(1)	
Cg(4) … Cg(1)^ii^	3.724(1)	

^i^ Related by symmetry operation: -x -y,2-z; ^ii^ Related by symmetry operation : -x,-y,2-z ^iii ^Related by symmetry operation: 1-x,-y,2-z.

### 2.4. X-Ray Diffraction Powder Analysis

**Figure 7:**
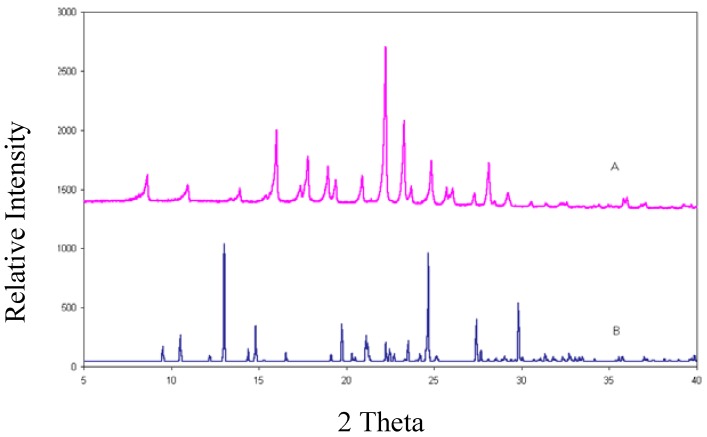
XRPD traces for rofecoxib (A) and **I** (B).

This is the most informative technique to establish whether a new crystalline phase has been created. If the XRPD (X-ray powder diffraction) trace of the material investigated differs from that of the reference material this implies that either a new polymorph has been formed or a chemical transformation has taken place. The XRPD trace (Figure 7) of rofecoxib is compared with the Lazy Pulverix [10] calculated trace for **I**. The completely different traces verify that a new crystal phase had indeed been created.

### 2.5. Mechanism

**Figure 8:**
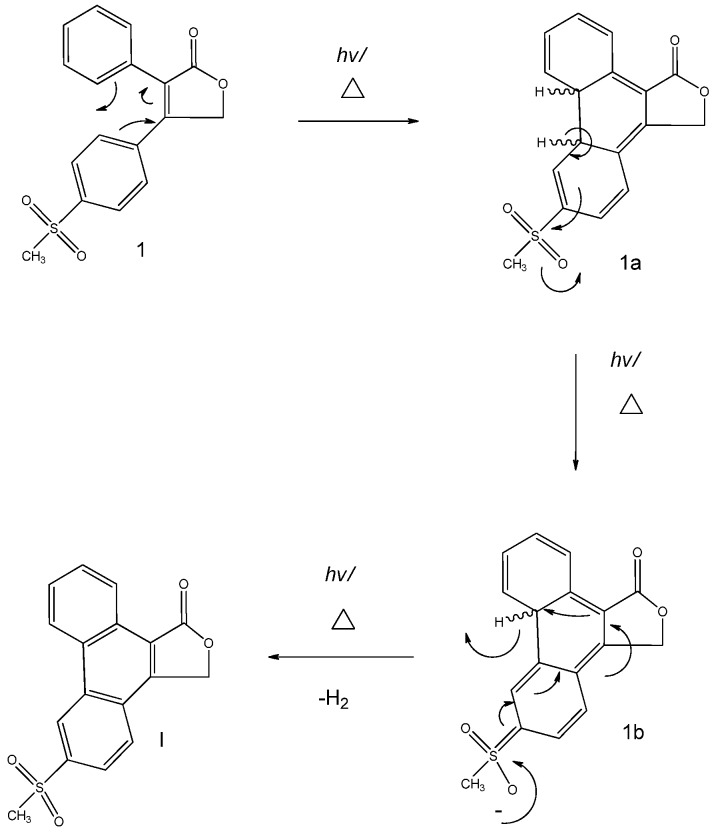
Mechanistic Scheme for the formation of **I**.

The starting material rofecoxib (**1**) formed the product **I** when various solvents (ethyl acetate and HPβCD solution) were used and when it was heated (65 °C) under room light intensity. A suggested mechanism is given in Figure 8. The class of pericyclic reactions that the mechanism corresponds to is electrocyclic, whereby one new σ bond forms across the ends of a single conjugated π system [11]. The stereo-outcome of this reaction is based on the Woodward and Hoffmann rules: the symmetry of the highest occupied molecular orbital (HOMO) controls the stereochemical outcome in thermal reactions and that of the lowest unoccupied molecular orbital (LUMO) controls the outcome of light induced reactions [11,12]. However, since the intermediate is not seen, the bond rotation of this 4n + 2 π electron system (see Figure 8) cannot be experimentally determined. Therefore reaction differentiation between heat and light is not known. 

However, the literature suggests that this is a photo-cyclization (light induced) product, implying that this has a conrotatory bond outcome [12,13,14]. The complimentary reaction that takes place thereafter is dehydrogenation *via* oxidation (**1a** and **1b**) ultimately forming the product **I**. The excellent anion-stabilising sulphone group provides the driving force behind aromatisation. This substituent is highly electron-withdrawing thereby promoting dehydrogenation. High temperatures favours dehydrogenation as the high activation energy required for this process is overcome by high temperatures. High temperatures also favour elimination by increasing the entropy in the free energy of the reaction. It could be tentatively hypothesized that the solvent could be acting as a poor nucleophile in aiding the primary dehydrogenation. Only the degradation product of rofecoxib, 6-methylsulphonyl- phenanthro-[9,10-C]-furan-1(3*H*)-one, was encountered in attempts to generate new forms of the drug. Nevertheless, strenuous stress trials conducted by Mao *et al.* [8] revealed that two major side-products may form. One of which results from the base promoted hydrolysis of the lactone moiety followed by oxidation, which yields a dicarboxylate; the other results from photo-cyclization of the *cis*-stilbene moiety which results in a phenanthrene derivative. In this investigation, the latter product was produced. 

## 3. Experimental Section

### 3.1. General

Hydroxypropyl-β-cyclodextrin (HPβCD) was purchased from Cyclolab (Budapest, Hungary) and used without further purification. The NSAID rofecoxib was sourced from Merck & Co Inc. (Rahway, NJ, USA) and used without pre-treatment. Water content was determined on fresh crystals by thermogravimetry (Mettler Toledo TGA/ISDTA851) under N_2_ purge (flow rate 10 mL/min) using samples of mass ~ 1 mg and a scan rate of 10 °C/min over the range 30−200 °C. DSC analysis was performed on a Perkin-Elmer PC7 system under N_2_–purge (flow rate 40 mL/min) using samples of mass ~5 mg and a scan rate of 10 °C/min. Reverse Phase HPLC were recorded on a Cintra 20 UV System with a detection wavelength of 280 nm using a variable wavelength UV detector. A 5 μg/mL, 1:1 stoichiometric ratio of rofecoxib: HPβCD solution and **I**: HPβCD solution was made and tested. Elemental analysis measurements using 2–3 mg of sample were performed on a Fisons EA1108 CHNS-O Elemental Analyser. NMR spectra were recorded on a Mercury VXR 400 Spectrometer using deuterated chloroform as the solvent and tetramethylsilane as the internal standard. PXRD: a microcrystalline sample of 5–10 mg was placed randomLy on a Mylar^® ^flat sample holder and intensities measured using a Huber Imaging Plate Guinier Camera 670. Nickel filtered CuKα_1_ radiation (λ = 1.5405981 Å) was produced at 40 kV and 20 mA by a Philips PW1120/00 generator fitted with a Huber long fine-focus tube PW2273/20 and a Huber Guinier Monochromator Series 611/15.

Compound **I** resulted from two methods, recrystallisation and co-precipitation. In the recrystallisation experiment 10 mg of rofecoxib was dissolved in 2 mL of ethyl acetate and stirred at 65 °C for 15 min. For the co-precipitation experiment 10 mg of rofecoxib was stirred in 2 mL 10% HPβCD solution at 60 °C for 72 h. All solutions were exposed to room light intensity. The resultant solution was thereafter filtered and left to evaporate at room temperature. Crystals formed after one month. ^1^H-NMR: δ H (200 MHz, CDCl_3_, Me_4_Si) 2.15 (s, 3H), 5.19 (s, 2H), 7.40 (t, 1H), 7.49 (t, 1H), 7.94 (d, 1H), 7.95 (d, 1H), 7.99 (d, 1H), 8.09 (d, 1H), 9.22 (d, 1H). EA: C 65.37, H 3.88, S 10.27%; required: C 65.38, H 3.92, S 9.79%. HPLC: Retention time 3.158 min. Mp onset: 307.1 ºC (Δ*H_f_* = 48.5 J/g).

### 3.2. Structure Determination and Crystal Data

CCDC 761742 contains the supplementary crystallographic data for this paper. These data can be obtained free of charge from The Cambridge Crystallographic Data Centre via www.ccdc.cam.ac.uk/data_request/cif. Intensity data were collected on a Nonius Kappa CCD diffractometer (1° frames in φ and ω, 2θ_max _= 56°, graphite-monochromated Mo-Kα X-rays, λ = 0.71069 Å, *T* = 173(2) K). Crystals were coated in Paratone oil (Exxon Chemical Co., TX, USA) immediately after isolation and cooled in a stream of nitrogen vapour on the diffractometer. Structures were solved by direct methods using the program SHELXS-97 [15] and refined using SHELXL-97 [15]. All non-hydrogen atoms were revealed in the E-map and subsequent difference electron density maps and thus placed and refined anisotropically. All H atoms were observed in difference syntheses and were placed in geometrically idealized positions and constrained to ride on their parent atoms with C־־־H distances in the range 0.95–1.00 A ˚ and Uiso(H) = xUeq(C), where x = 1.5 for methyl and 1.2 for all other C atoms. Crystal data for (**I**): C_17_H_12_O_4_S, M = 312.31, triclinic, space group P1, a = 8.4641(1), b = 9.2247(2), c = 9.4227(2) Ǻ, α = 79.8987(1), β = 85.9977(9), γ = 67.5502(2)º, U = 669.41(2) Ǻ^3^, Z = 2, Dc=1.5494 g cm^-3^, T=113(2) K, μ(MoKα) = 0.260 mm^-1^. Full-matrix least-squared refinement was based on 2679 reflection data and yielded wR2 = 0.1055 (all data), R1 [2333 data with F^2^ > 2σ(F^2^)] = 0.0475, and goodness-of-fit on F^2^ = 1.049.

## 4. Conclusions

A cyclized product resulting from the photo-degradation of rofecoxib was elucidated using thermoanalytical, elemental analysis, spectroscopic, chromatographic and crystallographic techniques. This unexpected product was obtained via recrystallisation and co-precipitation methods. The product was proven to be chemically different from rofecoxib *via* thermal analysis, HPLC, XRPD and elemental analysis. The structure of the product was determined by NMR spectroscopy and single crystal X-ray analysis. Following an extensive literature search it was discovered that this product had been revealed previously but not characterised to the extent reported in this paper. This form results from the photo-cyclization of the *cis*-stilbene moiety which results in a phenanthrene derivative, thus proving that rofecoxib is highly photo-sensitive. These results emphasise how proper control of the recrystallisation procedure of rofecoxib is of importance in order to maintain its structural veracity. In addition, as a part of future work, the biological effects of this degradation product should be investigated in order to ascertain its physiological impact.
